# Baltimore College of Dental Surgery

**Published:** 1844-09

**Authors:** 


### ARTICLE VI.

### BALTIMORE COLLEGE OF DENTAL SURGERY.

As every thing connected with the cultivation and advancement of the science and art of dental surgery, must be interesting to the profession, we publish below the Fifth Annual Announcement of this institution, and ask for it an attentive perusal.—*Eds.*

### VISITORS.

THOMAS E. BOND, SEN. M. D. *President.*
JOHN FONERDEN, M. D. *Secretary.*

| | |
|---|---|
| R. S. STUART, M. D. | SAMUEL CHEW, M. D. |
| JOSHUA I. COHEN, M. D. | REV. G. C. M. ROBERTS, M. D. |
| THOMAS C. RISTEAU, M. D. | J. J. GRAVES, M. D. |
| REV. JOHN G. MORRIS, | REV. J. P. K. HENSHAW, |
| REV. BEVERLY WAUGH, | REV. JAMES G. HAMNER, |
| JOHN H. BRISCOE, M. D. | LEONARD MACKALL, M. D. |

ENOCH NOYES, M. D.

### FACULTY.

CHAPIN A. HARRIS, M. D.
Professor of Practical Dentistry.

THOMAS E. BOND, JR M. D.
Prof. Special Pathology and Therapeutics.

W. R. HANDY, M. D.
Professor of Anatomy and Physiology.

C. A. HARRIS, M. D., and
THOMAS E. BOND, JR., M. D.
Profs. of Dental Physiology and Pathology.

J. B SAVIER, D. D. S.
Demonstrator of Mechanical Dentistry.

The fifth session of the Institution will commence on the first Monday in November next, and continue until the latter part of February.

The Faculty desire to urge upon the public, and especially upon the well informed Dentists, who are interested in the improvement of the Branch of Medicine to which this School is devoted, the claims which it has to their earnest and active support.

After four years' struggle to sustain this infant enterprize amidst general indifference, and against strong opposition, we feel that we may properly expect, that its pretensions will be carefully examined. If this be done, we cannot doubt that its support will be ample, and the result most gratifying to all who shall have aided in its early struggle for existence.

We are well aware, that any attempt to improve the condition of a profession, hitherto without regular organization; to erect a standard of excellence among men, before most unjustly confounded as equals, and to separate by a clear line of discrimination, the man of science from the empiric, must necessarily alarm many, and put them upon their defence against innovation so threatening. The man who encloses a field, long trodden as a common, will offend his neighbors, however just may be their exclusion. Against this feeling we have been prepared to contend, and though we have found it more extensive and powerful than we could have anticipated, we have, nevertheless, endeavored patiently to endure it.

To men, such as we have alluded to, we make no appeal. From them who are conscious of ignorance, long, artfully, and painfully concealed; who live upon a reputation obtained without merit, and liable to be destroyed in a moment by discovery of truth, from these, and such as these, we expect no aid, as from them we cannot hope for sympathy. But we call upon the men of worth; upon those who have long and keenly felt the mortification of being classed with the hordes of ignorant and impudent empirics who assume the name of Dentist, in the most prominent places of our cities, and in the obscure ale-houses of remote country-towns; we call upon those men who have secretly cultivated that professional pride which always accompanies professional knowledge, and we ask them to rally to the support of an Institution, the downfall of which would consign the Art of Dentistry, to the low place it now occupies, for at least fifty years to come.

For the information of all who may wish to know the constitution of the School, and the plan of instruction pursued in it, we subjoin the following statement:

1st. The College is chartered by the State of Maryland, with full powers to give Instruction, confer Degrees, &c.

2d. As scientific dentistry is but a branch of medicine, the College embraces a Medical and Dental Department—each of which is committed to two Professors—the first being Practitioners of Medicine, the latter of Dentistry.

3d. The course of instruction embraces the following named subjects:

1. Practical Dentistry,
2. Dental Physiology and Pathology,
3. Special Pathology and Therapeutics, including principles of Surgery,
4. Anatomy and Physiology.

*Practical Dentistry* includes both the *mechanical* and *operative department.* The most untiring efforts are devoted to teaching mechanical dentistry. A convenient work-room has been fitted up for this purpose, and the pupils have the advantage of making all the preparations and performing the manipulations under the eye of an experienced teacher. As it has been very incorrectly supposed that the education was merely theoretical, we desire, particularly, to call attention to this part of our announcement. To ensure excellence in this department, the faculty have secured the services of a skilful Mechanical Demonstrator, who will constantly afford instruction to the pupils while engaged in the work-room. Indeed, such facilities have been provided, and such stress is laid upon the importance of this department, that it must be the fault of the student himself if he does not become well versed in the mechanics of the dental art.

*Dental Physiology and Pathology.*—It is the province of the professor to describe the healthy and diseased conditions of the teeth, with the causes of the latter, and the modes of prevention. His lectures are illustrated by healthy and morbid preparations, models, &c.*

*Special Pathology and Therapeutics.*—The professor teaches the general principles of pathology, medicine and surgery. He dwells particularly upon such diseases as complicate disorders of the mouth, face, &c.

*Anatomy and Physiology.*—The professor teaches so much of general anatomy as is sufficient to familiarize the student with the elementary constitution of the human body. Then upon

* Until the vacancy made by the death of Professor Hayden, shall be permanently filled, which will be done as soon as possible, the duties of this chair will be divided. Professor Harris will deliver a special course of lectures on the *Pathology*, and Professor Bond on the *Physiology* of the Teeth, Mouth, &c.

the subject, he demonstrates, minutely, the important parts, dwelling in detail upon the anatomy of the head, neck, mouth, &c. In the demonstration of these parts, he will be aided by models, preparations, drawings, &c., of which an abundant supply is at his command.

The College possesses an excellent, well ventilated dissecting room, and abundant means of gaining practical knowledge in this most important department, are offered to the pupil, who is urged to use the knife freely as absolutely necessary for the acquirement of information necessary to the dentist who wishes thoroughly to understand his business.

Independently of his regular course, the professor delivers lectures on surgical anatomy, to a private class. Pupils of the Dental College, who may attend the dissecting room, will have the privilege of hearing these lectures without additional charge.

As it is the aim of the Faculty to make their several departments as practical as possible, frequent and close examinations upon the lectures are made by each professor.

The school has been in operation four years. It has been attended by thirty-four students, seventeen of whom have graduated and are in practice. Of these, several were gentlemen who had been in practice for years, and obtained reputation as dentists, but who were so convinced of the advantages offered by the school, as to become its pupils.

TERMS OF GRADUATION.

"Candidates for graduation who have attended two full courses of lectures in this college, or one course in some respectable medical college, and one in this institution, will be subjected to a critical examination by the Faculty, and be required to defend a thesis on some subject connected with dental science, they will also be required to present one or more specimens of mechanical skill in preparing and setting artificial teeth, and likewise be expected to perform certain dental operations in evidence of practical qualification; and on being found competent, they shall receive the degree of *Doctor of Dental Surgery.*"

### TERMS OF ADMISSION.

Ticket of each Professor, for each session, $25; Demonstrator's Ticket, $10; Dissecting Ticket, optional, $10; Diploma Fee, $30; Matriculation, $5.

Good and respectable board can be obtained at from $2 50 to $3 50 per week.

Lectures begin the first Monday of November and end the last of February.

### LIST OF GRADUATES.

| NAMES. | RESIDENCE. | THESES. |
|---|---|---|
| Robert Arthur, | Pennsylvania, | Dental Caries. |
| R. Covington Mackall, | Missouri, | First Dentition. |
| W. R. Scott, | North Carolina, | Dental Caries. |
| W. W. H. Thackston, | Virginia, | Diseases of the Maxillary Sinus. |
| Thos. B. Savier, | Virginia | Irregularities of the Teeth. |
| Charles Harris, | Maryland, | Dental Science, its Progress, Uses and Prospects. |
| John W. Foster, | Virginia, | Formation and Growth of the Permanent Teeth and the Destruction of the Roots of the Temporary. |
| B. R. Robinson, | Maryland, | The Mouth—Influence which Diseases of this Cavity exercise over other parts of the System. |
| C. G. Linthicum, | Maryland, | Diseases of Gums and Alveolar Processes. |
| Thos. G. Lockerman, | Maryland, | Dissertation on the Gums. |
| M. S. Robinson, | do. | Decay of the Teeth. |
| R. W. Thompson, | do. | Extraction of Teeth. |
| Thos. P. Lee, | S. Carolina, | Advantages of Artificial Teeth. |
| J. Wesley McGee, | Pennsylvania, | Spongy and Inflamed Gums, with Ab sorption of their Edges and Alveolar Processes. |
| Jno. W. Howlit, | N. Carolina, | Diseases of the Gums. |
| James Locke, | Pennsylvania, | Mineral Paste. |
| Wilson L. Hollifield, | do. | Importance of Plugging Teeth. |

